# Application of Magnetic Force Microscopy for Investigation of Epitaxial Ferro- and Antiferromagnetic Structures

**DOI:** 10.3390/ma10101156

**Published:** 2017-10-06

**Authors:** Gennady M. Mikhailov, Anatoliy V. Chernykh, Lev A. Fomin

**Affiliations:** Institute of Microelectronics Technology and High Purity Materials RAS, 142432 Chernogolovka, Russia; chern@iptm.ru (A.V.C.); fomin@iptm.ru (L.A.F.)

**Keywords:** exchange bias, magnetic force microscopy, epitaxial microstructures, Fe, FeMn

## Abstract

Growing of epitaxial Fe_50_Mn_50_/Fe/Mo/R-sapphire films was performed with a new configuration of two in-plane easy axes of Fe(001)-layer magnetization in which application of annealing in a magnetic field forms an unidirectional anisotropy. The microstructures made from these films exhibited an exchange bias 25–35 G along an exchange field generated at antiferromagnet/ferromagnet (AFM/FM) interface. Magnetic force microscopy (MFM) experiments supported by micromagnetic calculations and magneto-resistive measurements allowed interpretation of the magnetic states of the Fe layer in these microstructures. The magnetic states of the iron layer are influenced more by crystallographic anisotropy of the Fe-layer than by unidirectional exchange anisotropy.

## 1. Introduction

The phenomenon of exchange bias of the hysteresis loop in FM/AFM (ferromagnetic/antiferromagnetic) structures was discovered quite a long time ago [1,2]. However, at the present time, interest in this still persists in connection with practical applications. There are many theoretical models of this effect [3], which consider the interfaces between ferro- and antiferromagnet as either uncompensated [2] or compensated [4]. A large variety of magnetic ordering on frustrated FM/AFM interfaces is described in [5]. In any case, the quality of the interface influences the magnitude of the effect, motivating epitaxial growth of FM/AFM layers. It is known that antiferromagnetic Fe_50_Mn_50_ alloys are the most commonly used material in planar structures for implementing exchange anisotropy at the FM/AFM interface. Permalloys Fe_20_Ni_80_ with the same crystal structure as Fe_50_Mn_50_ face-centered cubic (FCC) and close lattice parameters are widely exploited as ferromagnetic layers. Cu or Ta [6,7] sublayers are commonly used as the buffer layers, as they contribute to textural growth of multilayered Fe_20_Ni_80_(111)/Fe_50_Mn_50_(111) films, even on disordered substrates.

Systems similar to FCC FM and AFM materials have been the most extensively investigated to date. In particular, the macroscopic domain states of ultra-thin permalloy films that have been exchange-biased by an FeMn layer have been studied in the literature [8,9], together with the kinetics of their magnetization and its correlation with the specific features of hysteresis loops. At the same time, epitaxial Fe(001)/Fe_50_Mn_50_(001) films have not been practically studied. One of the reasons for this may be insufficient matching of their lattice parameters. Indeed, the body-centered cubic (BCC) iron lattice parameter a = 0.287 nm can be fitted to the FCC Fe_50_Mn_50_ lattice parameter a = 0.363 nm only by a lattice rotating at 45° with an epitaxial mismatch of about 11%. Generally, non-epitaxial iron layers covered by antiferromagnetic layers have been studied. In particular, the methods of MFM (magnetic force microscopy), micromagnetic calculations and MOKE (magneto-optical Kerr effect) have been applied to investigate the magnetic states of ring-type microstructures [10]. Exchange bias and unidirectional anisotropy were found in these systems.

However, the influence of the crystallographic anisotropy of iron has not been investigated yet in AFM/FM structures, since the effect of anisotropy has been small in investigated structures. In epitaxial Fe(001) structures, there are two in-plane equivalent easy axes of magnetization directed along [100] and [010] [11], respectively, that create a new unstudied magnetic configuration, influenced by exchange anisotropy at the FM/AFM interface.

In this work, a magnetic force microscopy supported by magneto-resistance measurements and micromagnetic calculations has been applied to study the influence of exchange anisotropy on the magnetic state of the epitaxial FM layer in multilayered Fe_50_Mn_50_(80–150 nm)/Fe(40 nm)/Mo(5 nm)/R-sapphire microstructures. 

## 2. Experimental

Multilayered Fe_50_Mn_50_(80–150 nm)/Fe(40 nm)/Mo(5 nm)/R-sapphire films were grown by pulsed laser sequential layer-deposition in a vacuum of 10^−8^ torr on the monocrystalline sapphire substrates (R-plane) with a molybdenum sublayer. The substrate temperature during depositions was maintained at 400 °C for the Mo layer, and 280 °C for the Fe and FeMn layers demanding optimal epitaxial layer growth. The surface roughness of grown films measured by scanning atomic force microscope was characterized by a RMS (root mean square) of 0.5 nm and a roughness correlation length of 30–50 nm.

For MFM measurements, subtractive microstructurization of grown multilayered films was used for fabrication of discrete planar structures shaped as squares and rectangles with an aspect ratio of 1:2 of various (1–10 μm) sizes. The square and rectangle microstructures were oriented at different angles of 0° or 90° relative to the crystallographic direction of the Fe [100]. Subtractive technology included the etching of the grown films by argon ions, previously covered by aluminum micromasks that were fabricated on the surface of the films using electron lithography, aluminum deposition and the lift-off procedure. Aluminum micromasks were then removed with wet chemistry at the end of microstructure fabrication [12]. 

For magneto-resistive measurements, bridge-type macrostructures (200 μm × 800 μm) were fabricated by deposition through the mask in the same way used for the film growth procedure described above.

All samples were annealed in vacuum in a magnetic field of 1000 G applied along the Fe [010] axis for formation of unidirectional magnetic anisotropy. After annealing for one hour, the samples were slowly cooled down from a temperature of 250 °C, clearly exceeding the Neel temperature for Fe_50_Mn_50_ (160–230 °C), to room temperature. 

Magneto-resistive measurements of bridge-type structures were used to control exchange bias. An in-plane external magnetic field was applied along or perpendicular to the long axis of the bridge-type structures, and resistance measurements were carried out using a DC four-point scheme.

For the MFM measurements, a two line-scan modulated procedure (lift mode) was used. During the first line-scan, the topography of the microstructure was measured, and during the second one, the magnetic tip was repeatedly lift-off measured at a distance of 50 nm along the sample normal of the first line-scan topography during its movement along the surface. The magnetic tip oscillated at the resonant frequency of the cantilever level, and its phase was measured in the second line-scan. After completing the 2d scan, the measured phase was used as the 2d magnetic contrast image of the microstructure. A silicon cantilever (Tipsnano, Moscow, Zhelenograd, Russia) with a resonant frequency 180 kHz covered by 50 nm Fe-layer was used. The magnetic tip was magnetized along its axis, and its remagnetization field exceeded 100 G. In-plane external magnetic fields could be applied in the range from −100 to +100 G during the MFM measurements. 

## 3. Results and Discussion

### 3.1. Magnetoresistance Measurements

**Figure 1:**
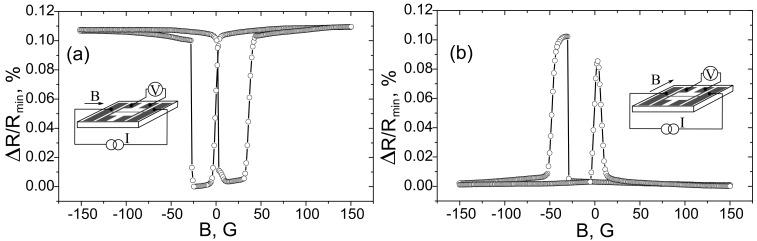
The dependence of the FeMn/Fe/Mo/R-sapphire bridge-resistance against an applied in-plane external magnetic field. It is perpendicular to the bridge in (**a**), and parallel to the bridge in (**b**). The magnetic field during the sample annealing procedure was always applied perpendicular to the bridge in the plane of the sample.

Magneto-resistive measurements of the bridge-type structures showed that the films Fe_50_Mn_50_/Fe/Mo/R-sapphire exhibit an exchange bias along the Fe [010] crystallographic direction, while Fe/Fe_50_Mn_50_/Mo/R-sapphire films do not. The dependence of the bridge resistance against applied in-plane magnetic field is shown in Figure 1. Figure 1a presents the curve of the longitudinal magnetoresistance (magnetic field along the bridge). This may be explained by an anisotropic magnetoresistance effect for the magnetic field applied parallel to the current. The curve is symmetric with respect to B = 0. The curve of transverse magnetoresistance (magnetic field perpendicular to the bridge) is presented in Figure 1b, demonstrating two peaks typical of an anisotropic magnetoresistance effect for a magnetic field perpendicular to the current. It can clearly be seen that the peak positions are displaced against B = 0 by 25 G to the left, manifesting an exchange bias.

Magnetoresistance curves could slightly vary from sample to sample; however, all experiments showed the appearance of an exchange bias along the direction of the magnetic field applied during sample annealing.

Thus, the magneto-resistive measurements showed that an exchange bias is observed in Fe_50_Mn_50_/Fe/Mo/R-sapphire bridge structures if the external magnetic field is along the Fe [010] crystallographic axis, coinciding with the direction of the magnetic field applied during sample annealing, but is not observed if the magnetic field is along the Fe [100] axis. Note that, if magnetic field applied during sample annealing was along the Fe [100] axis, the exchange bias was observed for longitudinal magnetoresistance but not for the transverse one. This indicates that the appearance of unidirectional anisotropy is due to the effect of the magnetic field applied during sample annealing. This is in agreement with [2], which models the uncompensated inner surface of the antiferromagnetic layer adjoining the ferromagnetic layer.

### 3.2. MFM-Experiments 

**Figure 2:**
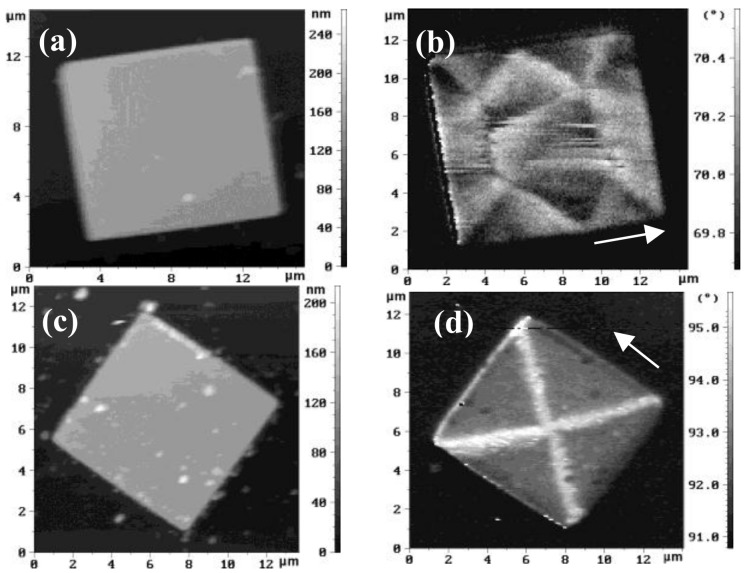
Topography and MFM images of square Fe/ Fe_50_Mn_50_/Mo/R-sapphire structures (**a**,**b**), and the same (**c**,**d**) for Fe_50_Mn_50_/Fe/Mo/R-sapphire structures. The arrows indicate the field direction during annealing.

MFM measurements revealed that, in the case where the FeMn layer was grown on the top of the Fe layer, the square microstructures had a regular magnetic state (Figure 2d). When the FeMn film was at the bottom, the magnetic structure was less regular (Figure 2b). The latter can be explained by the lower epitaxial quality of the samples due to the large mismatch between the crystal lattices of the Fe and FeMn layers. The iron layers are steadily grown on the epitaxial sublayer of molybdenum by cube-on-cube with a lattice mismatch of less than 10% (9.7%); the Fe_50_Mn_50_ layer on Fe(001) layer grows at a crystal rotation of 45° and a lattice mismatch of 11.8%. Epitaxial growth of the Fe_50_Mn_50_ layer on the Mo(001) sublayer breaks, however, because of the larger lattice mismatch of 13.2%.

The observed domain structures in the MFM for both cases were very different from each other. For Fe/Fe_50_Mn_50_/Mo/R-sapphire structures, the domains were located as if there were one axis of easy magnetization in the film plane perpendicular to the direction used during sample annealing in magnetic field (Figure 2b). Actually, this can be observed [3], if a FM/AFM interface is compensated. For Fe_50_Mn_50_/Fe/Mo/R-sapphire square structures, their magnetic state is quite different (Figure 2d). It consists of four triangle domains separated by ninety-degree domain walls. This is because there are two in-plane easy axes of magnetization, the same as in epitaxial iron films without a Fe_50_Mn_50_ layer on their top. This defines the kind of magnetic state that is realized. However, the investigated domain structure is slightly distorted in comparison with those of the epitaxial monolayered Fe(001) square microstructures [11,13] because of the presence of the exchange field at the Fe_50_Mn_50_/Fe interface. For comparison, topographical images of square mictrostructures are shown in Figure 2a,c. Additionally, some topographical features can be observed in MFM-images (Figure 2b,d) they obviously possess an entirely different nature from the measured magnetic contrasts of the magnetic states.

**Figure 3:**
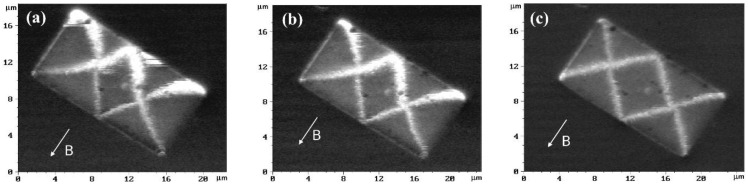

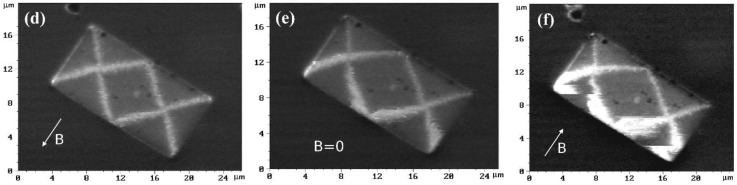
MFM images of rectangular microstructures in an external magnetic field: (**a**) −80, (**b**) −60, (**c**) −30, (**d**) −15, (**e**) 0 and (**f**) +15 G. Effective exchange field was formed along the short side of the rectangles. The arrows indicate an external magnetic field. The vertical scale is the phase shift and ranged from 0° (black) to 2° (white).

**Figure 4:**
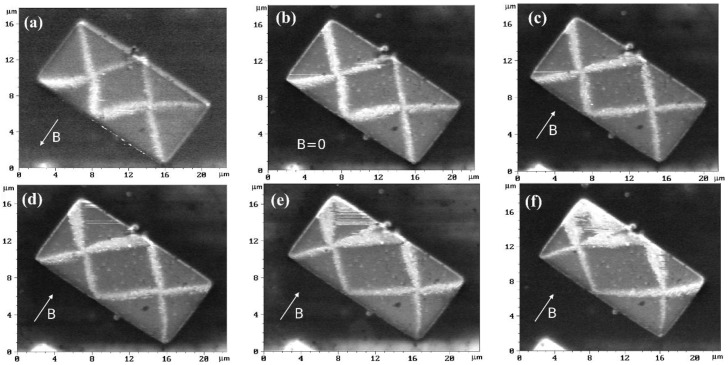
MFM images of rectangular microstructures in an external magnetic field: (**a**) −15, (**b**) 0, (**c**) +15, (**d**) +30, (**e**) +45 and (**f**) +60 G. Effective exchange field was formed along the long side of the rectangles. The arrows indicate an external magnetic field. The vertical scale is the phase shift and ranged from 0° (black) to 2° (white).

MFM experiments in the presence of an external magnetic field allowed measuring this built-in field without application of electric contacts. It is more convenient to use rectangular Fe_50_Mn_50_/Fe/Mo/R-sapphire microstructures in these experiments. MFM images of them in an external magnetic field, oriented along the short side of the rectangle are shown in Figure 3 and Figure 4. The built-in exchange field in the magnetic field, which originated during the annealing procedure, was directed in turn along the short and long sides of the rectangle.

**Figure 5:**
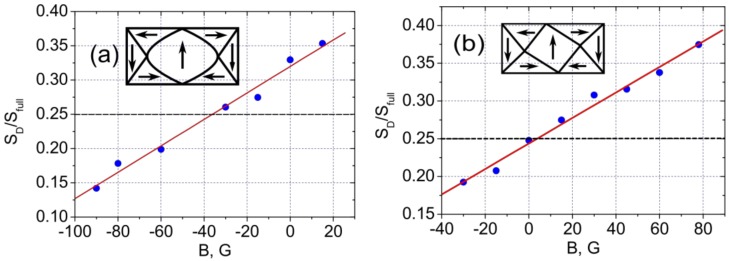
The dependence of the central domain of the rectangle areas ratio against the applied magnetic field. The magnetic field is directed along (**a**), and perpendicular (**b**) to the exchange built-in field. The inserts schematically show the domain structure in zero magnetic fields. The spatial distribution of magnetization and its direction have been taken from the micromagnetic calculations (see below).

It is known that the magnetic state of a rectangular microstructure of Fe(001) with an aspect ratio 1:2 is of “diamond” type [13]. At zero magnetic field, the area of the central domain is equal to 1/4 of the area of the whole rectangle. If there is a built-in exchange field directed along the magnetization of the central domain, the central domain will be widened. This broadening is most clearly seen in Figure 3c. Graphs of the normalized central domain area of the rectangles corresponding to Figure 3 and Figure 4, are presented in Figure 5a,b, respectively. Approximation of the experimental data of a normalized central domain area (Figure 5a) gives a value for the exchange bias of about minus 35 G for the structures that, during annealing, were magnetized perpendicular to the long side (Figure 3). The value of an exchange bias was found at the point where the curve crosses 1/4*.*

This value is close to the exchange bias found from magnetoresistance measurements. The difference between data can be explained by the non-equal lateral dimensions of the structures used in these experiments and possible influence of electric contacts applied in magnetoresistance measurements. Approximation of experimental MFM data gave a value of zero for exchange bias (Figure 5b) for structures that during annealing was magnetized parallel to the long side.

### 3.3. Micromagnetic Calculations

To explain the experimental results of MFM Fe_50_Mn_50_/Fe/Mo/R-sapphire structure investigation using a model that considers an uncompensated inner surface of the antiferromagnet at the FM/AFM interface, micromagnetic calculations were carried out. OOMMF [14] software (version 1.2a5) was used. Calculations were performed for a rectangular Fe/FeMn microstructure of 1 × 2 μm^2^ lateral dimensions. The thickness of the iron layer was assumed to be 40 nm. The size of the calculation cell was 5 × 5 × 5 nm^3^. The antiferromagnetic layer was modeled by two oppositely spin-directed elementary layers, the thickness of each layer was equal to cell size (5 nm) and their spins were considered frozen during calculation. Spins in one layer were oriented in a common direction, thus forming an uncompensated surface at the AFM/FM interface. The saturation magnetic moment was taken to be M_s_ = 800 emu for FeMn and M_s_ = 1700 emu for Fe. The exchange stiffness for Fe was A = 21 × 10^−7^ erg/cm. The anisotropy of Fe was considered to be cubic with in-plane easy axes of magnetization along [100] and [010] directions. The constant of cubic anisotropy for Fe was 4.8 × 10^5^ erg/cm^3^. The exchange and anisotropy parameters of FeMn layers were not important in the calculations, because the spins of the layers were frozen. The constant of exchange interaction between the Fe- and FeMn-layers at their interface was assumed to be equal to 1 × 10^−7^ erg/cm, which is much lower than the exchange stiffness of Fe. This can be considered an effective (fitting) parameter, because the applied model disregards both modification of the interface magnetic structure and interface roughness. The RMS of the grown films is about 0.5 nm, which exceeds the distance between elementary layers of AFM magnetic sublattices. As a result, the roughness may have a significant effect on the exchange interaction between AFM and FM layers on their interface. 

During calculations, a simulated external magnetic field was always directed along the short side of the rectangle, while the spin direction in the uncompensated antiferromagnetic layer at the AFM/FM interface was along the short or long rectangular side. For calculation, the initial conditions of the Fe layer magnetic state in the bilayered Fe/FeMn rectangle was was selected as a “diamond” magnetic state, which is typical of monolayered Fe-rectangles without an external magnetic field. In this approach, if the found simulated magnetic contrast and the sign of exchange bias coincide with the experimental results, it is assumed that the calculated magnetic state represents the magnetic state of the real microstructure.

**Figure 6:**
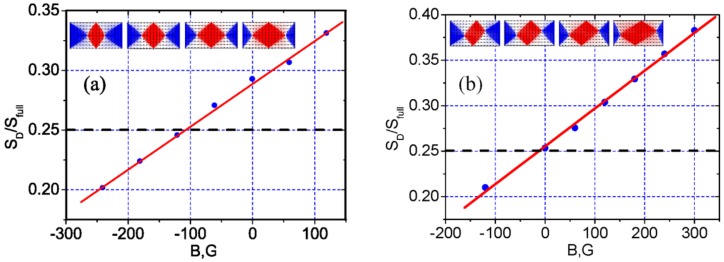
The calculated dependence of the central domain on the rectangle areas ratio against applied magnetic field. The exchange built-in field is perpendicular (**a**) or parallel (**b**) to the long side of the rectangle. The inserts display the calculated magnetic states of the Fe layer in magnetic fields −300, −120, 0, 120 (**a**) and −120, 0, 120, 300 G (**b**).

The results of the calculations are presented in Figure 6. Figure 6a shows the normalized (to the rectangle area) area of the central domain of the magnetic state as a function of the simulated external field, where the directions of the external magnetic field and the antiferromagnetic-layer spin coincide at the FM/AFM interface. An exchange bias of about 100 G can be clearly seen. Figure 6a shows the same, when the simulated external magnetic field is perpendicular to the ferromagnetic layer spin direction. No exchange bias is observed in this case. The inserts represent how the “diamond” magnetic state of the Fe-layer in the bilayered FeMn/Fe rectangle is transformed by the external magnetic field and unidirectional anisotropy. Note that the central domain is symmetrically distorted (Figure 6a) if the built-in exchange field and external magnetic field are parallel to each other, and asymmetrically (Figure 6b) if they are perpendicular. This is because the effective field, which is a vector sum of the built-in exchange field and external magnetic field, may be parallel or not to the wall-side of the microstructures. The same is observed experimentally, if the MFM magnetic contrasts in Figure 3 and Figure 4 are compared.

On a few occasions, the exchange bias obtained from the calculations exceeded the experimental one. A possible explanation of this discrepancy could be the presence of structural defects in the experimental samples; in particular, roughness at the AFM/FM interface that was not taken into account during the calculations.

Micromagnetic calculations are semi-quantitatively consistent with the MFM experiments, and support the uncompensated surface model for the case, when FeMn layer is on the top of the Fe-layer. For the opposite case, if the Fe layer is on the top of the FeMn layer, it may be assumed that the FeMn layer forms a compensated surface at the FM/AFM interface.

## 4. Conclusions

Growing of epitaxial bilayered Fe_50_Mn_50_/Fe/Mo/R-sapphire films of high quality has been performed with a new configuration of two in-plane easy axes of Fe(001)-layer magnetization in which application of annealing in a magnetic field formed a unidirectional anisotropy arising from the exchange interaction at the AFM/FM interface. It was shown that the microstructures made from these films exhibit an exchange bias of 25–35 G along the exchange field generated at the AFM/FM interface. MFM experiments supported by micromagnetic calculations and magneto-resistive measurements allowed interpretation of the magnetic states of the Fe layer, and its dependence on external magnetic fields and unidirectional anisotropy. The magnetic states of the iron layer are influenced greatly by the crystallographic anisotropy of the Fe-layer with close to the magnetic state of monolayered Fe-microstructures magnetization distribution, and to a lesser extent, by unidirectional anisotropy. The latter results only in small distortion of the magnetic state. 

Micromagnetic calculations semi-quantitatively confirm the model of a spin-uncompensated Fe_50_Mn_50_/Fe interface formed by an FeMn layer sited on the top of the Fe layer, while interaction between FM and AFM layers in Fe/Fe_50_Mn_50_ microstructures can be modeled as compensated one.
